# The Changes in Color, Soluble Sugars, Organic Acids, Anthocyanins and Aroma Components in “Starkrimson” during the Ripening Period in China

**DOI:** 10.3390/molecules21060812

**Published:** 2016-06-22

**Authors:** Yulian Liu, Nianlai Chen, Zonghuan Ma, Fei Che, Juan Mao, Baihong Chen

**Affiliations:** 1 College of Horticulture, Gansu Agricultural University, Lanzhou 730070, China; yulianliu@126.com (Y.L.); mazohu@163.com (Z.M.); maojuan@gsau.edu.cn (J.M.); 2 College of Resources and Environmental Sciences, Gansu Agricultural University, Lanzhou 730070, China; chennl@gsau.edu.cn; 3 Sichuan Kaijiang Middle School, Dazhou 626350, China; aprol_cf@163.com

**Keywords:** apple, color, anthocyanin, soluble sugar, organic acid

## Abstract

“Starkrimson” is a traditional apple cultivar that was developed a long time ago and was widely cultivated in the arid region of the northern Wei River of China. However, little information regarding the quality characteristics of “Starkrimson” fruit has been reported in this area. To elucidate these characteristics, the color, soluble sugars, organic acids, anthocyanins and aroma components were measured during the ripening period through the use of high performance liquid chromatography (HPLC) and gas chromatography-mass spectrometry (GC-MS). The results indicated that the changes in anthocyanin contents took place later than the changes in the Commission International Eclairage (CIE) parameters. Meanwhile, cyanidin 3-galactoside (cy3-gal), fructose, sucrose, glucose and malic acid were the primary organic compounds, and 1-butanol-2-methyl-acetate, 2-hexenal and 1-hexanol were the most abundant aroma components in the skin. Furthermore, rapidly changing soluble sugars and organic acid synchronization took place in the early ripening period, while rapidly changing aroma components occurred later, on the basis of fresh weight. This result suggested that the production of aroma components might be a useful index of apple maturity.

## 1. Introduction

Apples refill the reserves of vitamins, minerals, phenolic compounds, amino acids, dietary fiber and trace elements in the human body [1,2,3]; therefore, they have been a highly popular snack for a long time throughout the world. The color, soluble sugars and organic acids are important components of fruit taste, and together with the aroma, they have a strong impact on the overall organoleptic quality of fruits [4]. Currently, red-fleshed apples are receiving increased attention because of their remarkable anthocyanin contents, as it has been reported that anthocyanin contributes more to hydrogen peroxide scavenging than other phenolic compounds in the apple peel [5], and they are very important natural flavonoid compounds for the apple color [6,7]. Cyanidin 3-galactoside (cy3-gal) was identified as the major pigment in apples, accounting for 94% of the total anthocyanin content, and the contents of other anthocyanins, such as cyanidin 3-glucoside (cy3-glu), cyanidin 3-arabinoside (cy3-ara), cyanidin 3-rutinoside (cy3-rut) and cyanidin 3-xyloside (cy3-xyl) were very small [8]. The concentrations of the anthocyanins, especially cy3-gal, increased during maturation and ripening [6,9,10], coinciding with a corresponding increase in the percent of red blush [11,12].

The sugar content of apples differ depending on the cultivars, weather conditions, and culture technology as well as the position and exposition of the fruits in the crown [13,14]. The sugars consisted of fructose, glucose, sucrose, sorbitol and so forth, while the major organic acids in the apples included malic acid, succinic acid, oxalic acid and tartaric acid [12,15]. During the development of the apple fruit, the accumulation rates of sugars and acids were different. Sugars and sugar alcohols are synthesized and accumulate at a faster or similar rate relative to the growth of the apple, whereas organic acids are synthesized and/or accumulate at a slower rate relative to the apple growth [15]. The taste of apple fruit depends on the contents and types of soluble sugars and organic acids because fructose, glucose, and sucrose differ significantly in sweetness [16,17], and similarly, malic, citric, and tartaric acids are not equally acidic. In addition, the aroma components are an important quality factor in apples. A low production of volatile aromas was observed in early harvested fruit, which gradually increased as ripeness was approached [18].

“Starkrimson” is a black red apple cultivar. It was developed a long time ago but is still widely planted in some regions. In the arid area of the northern Wei River of China, “Starkrimson” is a very popular apple cultivar due to its deep red color, large fruit, appropriate sugar:acid ratio and well-balanced aroma, which all have a strong impact on the overall organoleptic quality of the fruit [4]. HPLC assessment of the anthocyanin, organic acid and sugar compositions has thus far been conducted for only a limited number of varieties, and the results indicate that the anthocyanin, sugar and organic acid accumulation in apples has three distinct characteristics. First, apples are rich in cy3-gal, which accounts for 80%–94% of the total anthocyanin content [9,12]. The second characteristic is fructose, which accounts for 44%–75% of the total sugars [19]. Last but not least, malic acid is the dominant acid in apple fruits, accounting for up to 90% of the total organic acids [15,19]. Therefore, more detailed knowledge of the anthocyanin, sugar and organic acid compositions and accumulation in different apple cultivars is still needed because it will provide further sufficient and beneficial information on metabolite composition, and the appearance of these characteristics largely depends on the degree of maturity and geographical location of different cultivars. Thus far, we have little information about the quality characteristics of “Starkrimson” fruit in the arid area of the northern Wei River of China. The purpose of this study was to investigate the development and changes in color, anthocyanins, soluble sugars, organic acids, and aroma components of “Starkrimson” fruit to understand the developmental metabolite patterns and to provide information concerning the composition and concentrations of metabolites to processors and consumers of this cultivar.

## 2. Results

### 2.1. Variation of Color and Anthocyanin Composition in Apple

**Figure 1:**
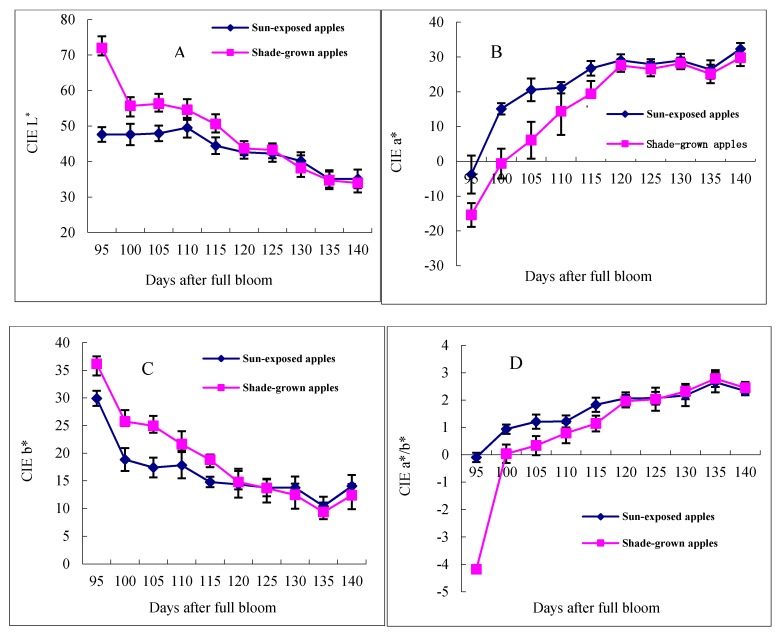
CIE parameters of “Starkrimson” during the ripening period.

The chromatic characteristics of the “Starkrimson” apple during the ripening period are shown in Figure 1. The changes in CIE L*, b* and a*, and a*/b* values varied differently at different number of days after full bloom on the exposed and shade sides of the apples. The L* and b* values decreased, while the a* and a*/b* values increased on both sides of the apples from 95 to 140 days after full bloom (DAFB). The L* value of the shade side was 64.11, while that of the exposed side was 49.66 on 95 DAFB, with the difference between them being 14.45. Then, the L* values of the shade side increased continuously until 125 DAFB, and from 125 to 140 DAFB, the L* values of the exposed side were the same as shade-grown apples. The results showed that the L* values of the exposed side gradually became closer to those of the shade side from 95 to 125 DAFB. Meanwhile, the b* value was 32.58 on the shade side on 95 DAFB, and it was 15.74 higher than that of the exposed side. Until 120 DAFB, the b* values of the shade side were always higher than those of the exposed side, and from 120 to 140 DAFB, the b* values of the exposed side were the same as those of the shade side. Interestingly, the b* values increased on both sides of the apples in the last five days.

For the other two indicators, the values of a* and a*/b* increased on both sides of the apples during the ripening period. The CIE a* is negative for green and positive for red. The a* values of the exposed side (−3.81) were higher than those of the shade side (−15.45) in “Starkrimson” apples. The a* values increased rapidly with time before 120 DAFB, and then they increased slowly. The a*/b* values increased from 95 to 120 DAFB. Therefore, from 95–120 DAFB is a very important stage for “Starkrimson” apple coloring because of the rapid changes in the values of a*, b* and a*/b* on both sides of the apples during this stage.

**Figure 2:**
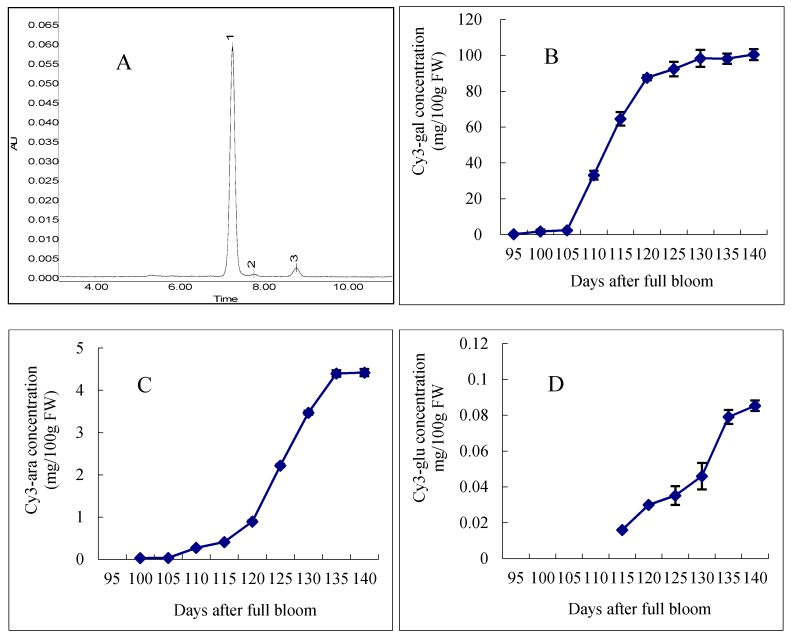
HPLC separation and concentration changes of anthocyanins in “Starkrimson” apple peel during ripening period. (**A**) “Starkrimson” apple peel monitored at 530 nm. Peaks: 1 cyanidin 3-galactoside; 2 cyanidin 3-glucoside; 3 cyanidin 3-arabinoside; (**B**–**D**) concentration and changes of cy3-gal, cy3-ara and cy3-glu in “Starkrimson” apple peel during ripening period and values are means ± SE (*n* = 4).

Analysis of the anthocyanin composition and concentration in the peel of samples harvested at different stages of ripening was carried out by HPLC (Figure 2). The HPLC chromatograms of apple extracts obtained at 530 nm revealed three peaks, which corresponded to the following three types of anthocyanins: cy3-gal (peak 1), cy3-glu (peak 2) and cy3-ara (peak 3). During the ripening period, the red blush of the fruit significantly increased, and the three anthocyanins cy3-gal, cy3-ara and cy3-gul were responsible for this change. Interestingly, different anthocyanin components appeared at different times. Among these three classes of anthocyanins, cy3-gal was the most abundant, and it was the first anthocyanin that appeared in the apple peel. From 95 to 105 DAFB, the content of cy3-gal increased slowly, followed by a rapid increase until 130 DAFB, with the content increasing from 2.43 to 98.33 mg/100 g FW (fresh weight). Meanwhile, from 130 to 140 DAFB, the cy3-gal content increased slowly. For cy3-ara, which was synthesized later and in a smaller amount, its content increased slowly from 95 to 105 DAFB. Then, it increased rapidly from 0.33 to 4.56 mg/100 g FW until 135 DAFB. The last synthesized anthocyanin component cy3-glu, which had the smallest content in apple skin, appeared 115 DAFB and increased rapidly until 140 DAFB.

### 2.2. Sugar and Organic Acid Contents of Apples

**Figure 3:**
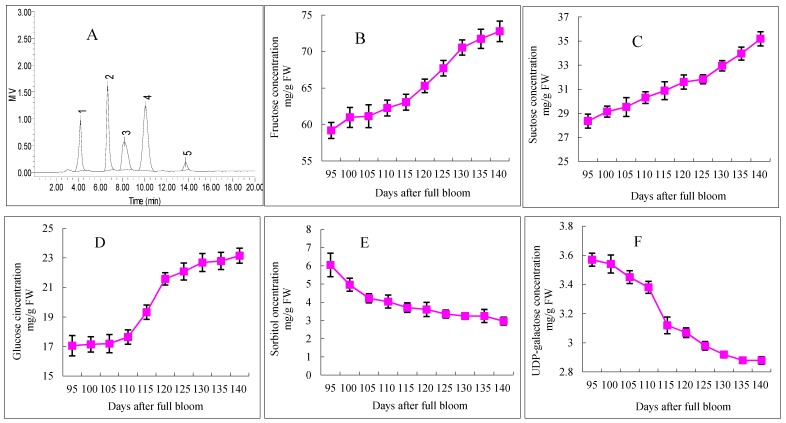
HPLC separation and concentration changes of soluble sugars in “Starkrimson” apple flesh during ripening period. (**A**) “Starkrimson” apple peel monitored by refractive index (RI) detector. Peaks: 1 UDP-galactose; 2 fructose; 3 sucrose; 4 glucose; 5 sorbitol; b, c and d values are means ± SE (*n* = 4); (**B**–**F**) concentration and changes of fructose, sucrose, glucose, sorbitol and UDP-galactose in “Starkrimson” apple peel during ripening period.

Five different sugars of “Starkrimson” apples were measured by HPLC, including uridine diphosphate-galactose (UDP-galactose), fructose, sucrose, glucose, and sorbitol. The variations in the concentrations of individual soluble sugars in “Starkrimson” during fruit ripening are shown in Figure 3. In the fresh weight, the concentrations of individual soluble sugars showed different changes. The concentrations of glucose, fructose, and sucrose generally increased during the ripening period, but the concentrations of sorbitol and UDP-galactose decreased. The concentrations of fructose and glucose increased slowly from 95 to 110 DAFB, and those contents increased from 59.19 to 62.26 mg/g FW and 17.19 to 17.65 mg/g FW, respectively. Then, both of these sugars increased rapidly from 110 to 130 DAFB, with the contents increasing from 62.26 to 70.55 mg/g FW and from 17.65 to 22.69 mg/g FW, respectively. After that, the fructose and glucose concentrations increased slowly until 140 DAFB. Moreover, the sucrose concentration did not show a slow-rapid-slow accumulation in “Starkrimson” apples, and it continuously increased until 140 DAFB from 28.36 to 35.19 mg/g FW. During the ripening period, the contents of sorbitol and UDP-galactose decreased. The highest concentrations of both sorbitol and UDP-galactose appeared at 95 DAFB, and then, they decreased from 6.05 to 2.97 mg/g FW and from 3.57 to 2.88 mg/g FW, respectively.

Significant positive correlations between the cy3-gal and the concentrations of sucrose and organic acids were observed (Table 1). The results showed that there were significant negative correlations between the UDP-galactose, succinic acid, acetic acid and cy3-gal concentrations, and a significant positive correlation between the sucrose and cy3-gal was found in this study.

**Figure 4:**
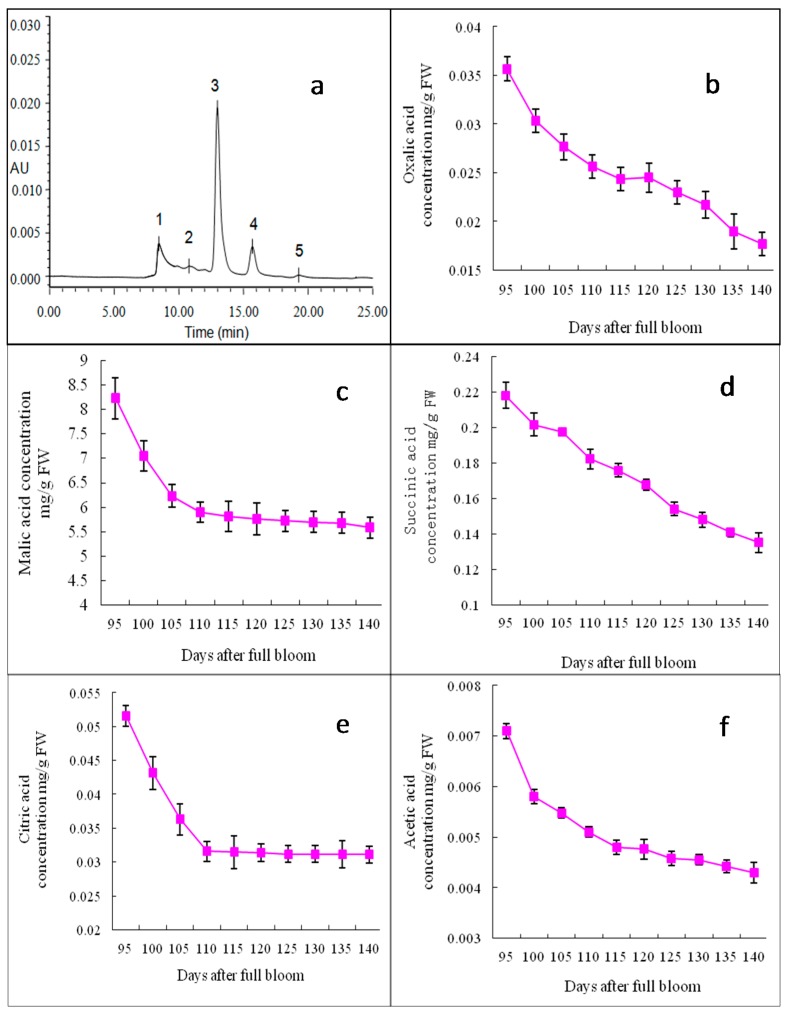
HPLC separation and concentration changes of organic acids in “Starkrimson” apple flesh during ripening period. (**a**) “Starkrimson” apple peel monitored by photo-diode array(PAD) detector. Peaks: 1 oxalic acid; 2 citric acid; 3 malic acid; 4 succinic acid; 5 acetic acid, and values are means ± SE (*n* = 4); (**b**–**f**) concentration and changes of oxalic acid, citric acid, malic acid, succinic acid, 6 acetic acid in “Starkrimson” apple peel during ripening period.

Oxalic acid, citric acid, malic acid, succinic acid and acetic acid were detected in “Starkrimson” apples, and there were great differences in the concentrations of individual organic acids, while the tendency was the same during the ripening period. Figure 4 shows the electropherogram and concentration changes of organic acids in the “Starkrimson” apple flesh. Malic acid was the main organic acid in the “Starkrimson” apples, and the contents of other organic acids were smaller. The concentration of malic acid decreased from 8.23 to 5.58 mg/g FW during 95–140 DAFB, but from 95–110 DAFB, they decreased rapidly from 8.23 to 5.90 mg/g FW. The trends for changes in the contents of citric acid and acetic acid were quite similar to that of malic acid. The oxalic acid and succinic acid contents continuously decreased.

#### Aroma Component Contents of Apples

In apples of different maturities, the contents and compositions of the aroma components were different. Thirteen different aromatic components were synthesized in “Starkrimson” fruit (Table 2) on 95 DAFB. C6-aldehydes, including 2-hexenal and hexanal, were the main aroma components accounting for 81.83% of the total volatile aroma. In addition, 2-hexen-1-ol acetate and acetic acid hexyl ester aroma components account for 7.31% of the total volatile aroma. On 115 DAFB, there were 30 aroma components in the apples, with aldehydes accounting for 42.96% of the total volatile aroma. The ester and alcohol contents increased, and the relative contents were 2.47 times and 2.98 times those at 95 DAFB, respectively. Some volatile aromas increased with ripening, including as 2-hexenal, (*E*)-2-hexenal, 2-hexen-1-ol acetate, hexanal, and acetic acid hexyl ester. Moreover, some new volatile aromas were detected such as 1-penten-3-ol, 1-butanol, 2-methyl butanol and 2-methyl acetate. Furthermore, on 125 DAFB and 135 DAFB, 43 and 53 volatile aromas were detected, respectively, with the esters increasing rapidly and accounting for 18.89% and 29.26% of the total volatile aromas. In addition, 2-hexenal, (*E*)-2-hexen-1-ol, hexanal, 2-hexen-1-ol acetate, and 1-butanol-2-methyl acetate showed higher contents among the volatile aromas. During this time, some new volatile aromas were also detected, such as hexanoic acid, hexyl ester, butanoic acid-2-methyl-hexyl ester, hexanoic acid hexyl ester, hexanoic acid butyl ester and butyl-2-methylbutanoate. On 140 DAFB, there were 36 esters among the 58 volatile aromas, accounting for 38.54% of the total volatile aroma, with aldehydes and alcohols accounting for 42.62% and 15.55%, respectively. Some volatile aromas had higher contents, such as 2-hexenal, hexanal, 1-butanol-2-methyl-acetate, 1-hexanol, acetic acid and hexyl ester, and some new volatile aroma were detected including propanoic acid butyl ester, *n*-propyl acetate, ethyl propanoate, butanoic acid butyl ester, α-curcumene and ethyl cis-3-hexenoate. Meanwhile, other volatile aromas were not detected such as (*E*)-2-hexen-1-ol and 3-hexen-1-ol, which had been measured before.

## 3. Discussion

### 3.1. Color and Anthocyanin Composition in Apple

In apples, the pigments responsible for color are chlorophyll, carotenoids and anthocyanins. Their formation is absolutely light dependent and is also affected by temperature [20,21,22]. Color aspects including the L*, a*, b* and a*/b* values reflect the same or related changes to the pigments [23]. During the fruit ripening, color parameters showed a characteristic tendency. The color parameters a* and a*/b* of partially ripe fruits were lower than those in ripe fruits, but the L* and b* values were higher [8]. In this study, the a* and a*/b* values increased, and the L* and b* values decreased on both sides of the apples during “Starkrimson” ripening periods. During the 95–120 DAFB stage, the values of L*, a*, b* and a*/b* in the apples changed quickly on both sides, which showed that this stage was the important stage for “Starkrimson” apple coloring.

Anthocyanins are the main pigments responsible for the color of apples, and as shown above, the three classes, cy3-gal, cy3-glu and cy3-ara, were responsible for the red color of apple peel. In previous studies, cy3-gal was reported to be the most abundant anthocyanin in red cultivars [24,25]. Other anthocyanins, such as cy3-glu and cy3-ara, were found in minor amounts in other apple cultivars [26,27]. It was found that the anthocyanins in “Starkrimson” apple peel were similar to those in other apple cultivars, with the only difference being in their contents. In general, the anthocyanin content increased quickly from 105 to 130 DAFB. Interestingly, we found that the changes in anthocyanin contents took place later than the changes in the CIE parameters, probably resulting from the influences of the chlorophyll and carotenoid contents in the early stages of apple coloring.

### 3.2. Changes of Sugar and Organic acid Contents of Apples

Soluble sugars and organic acids were important components in apples, influencing their taste. In mature “Starkrimson” apples, fructose, glucose and sucrose were the major soluble sugars, and sorbitol was the major sugar alcohol (Figure 3), which was consistent with the results obtained on other apple varieties in previous studies [15,28,29]. The similar developmental patterns in the glucose and fructose contents during the “Starkrimson” apple ripening period suggest that the synthesis and accumulation of glucose were closely linked with that of fructose. There was little information available on the concentration of UDP-galactose changes in apples. UDP-galactose may be enzymatically and chemically solubilized and degraded [30,31]. The metabolic mechanism of UDP-galactose still needs to be clarified. Significant negative correlations between the UDP-galactose, sorbitol, succinic acid, acetic acid and cy3-gal concentrations were observed (Table 1). In apples, sorbitol is converted to fructose, whereas sucrose was metabolized to glucose and fructose or UDP-glucose and fructose [26]. In this study, as the apples ripened, the increases in the fructose and glucose contents corresponded with a decrease in the sorbitol content. Malic acid was the main organic acid in mature “Starkrimson” apples, and other organic acids were found in minor amounts in other apple cultivars, which was consistent with other studies [15,29].

Moreover, soluble sugars and organic acids also play an important role in fruit coloration. Soluble sugars were the anthocyanin donor substrates and acted as signal molecules in the anthocyanin system [32]. Organic acids could serve to stabilize anthocyanins [33]. In this study, significant positive correlations between the glucose, fructose, and sucrose concentrations and cy3-gal were observed, especially sucrose with a correlation coefficient of 0.974 (Table 1). In a previous study, the anthocyanin content showed significant correlation with the concentration of sucrose in red and non-red apples [12], and several workers also stressed the importance of sugars for anthocyanin formation [23,34]. In this study, significant positive correlation between the organic acids and anthocyanins was shown (Table 1). However, this mechanism was equivocal and requires further demonstration.

### 3.3. Aroma Component Contents of Apples

“Starkrimson” is a popular apple cultivar due its deep red color, large fruit type, appropriate sugar:acid ratio and well-balanced aroma. The fruit flavor complex is mainly composed of volatile compounds that include a broad group of metabolites [35]. The aroma properties of fruit depend upon the combination of volatiles as well as the concentrations and thresholds of individual volatile compounds. In apples, the typical aroma compounds were the fruity esters that develop during ripening with a maximum endogenous ester concentration occurring at the climacteric peak [36,37]. Multivariate analysis showed that these compounds had the largest influence on the differentiation of stages of maturity. During “Starkrimson” apple ripening, the esters and alcohols increased while the aldehydes decreased. During 115–140 DAFB, the varieties of volatiles increased rapidly from 30 to 53, especially esters, which increased from 15 to 50, suggesting that the period from 115 to 140 DAFB was an important period for the formation of aroma compounds.

## 4. Materials and Methods

### 4.1. Plant Material

Apples were harvested from 5- and 6-year-old trees grafted on M26 rootstock and planted in the Northwest A & F University apple experimental station in Baishui County, Shanxi province, China. The planting density of the trees was 3 m × 1.5 m. The fruits were picked from 90 DAFB to 140 DAFB. Every 5 days, 10 apples were randomly selected among the 10 apple trees for measurements of the skin color as well as the accumulation of anthocyanins, soluble sugars, organic acids and aroma components using a colorimeter, ultraviolet spectrophotometer and HPLC, refractive index (RI) detector and HPLC, head-space solid phase microextraction (HS-SPME) and gas chromatography-mass spectrometry (GC-MS). After color measurements, the peels from the apples were collected in plastic bags, frozen and kept at −20 °C until extraction. The apple flesh was used to measure the sugars and organic acids. The fruit flesh was packed in plastic bags and kept at −80 °C until extraction. The other apple fruits were picked for extraction of the aroma components. The experiments were repeated four times with similar results.

### 4.2. Fruit Color Measurement

The apple color was measured by a Chroma Meter CR-400 chromaportable colorimeter (Konica Minolta, Tokyo, Japan) on exposed and shaded sides of each apple. The chromaticity of the fruit was recorded in the CIE parameters L*, a* and b* color space coordinates. The colorimeter was calibrated with a white standard calibration plate before use. In this system of color representation, the L* value corresponds to a dark-bright scale and represents the relative lightness of colors with a range from 0 to 100 (0 = black, 100 = white). The values of a* and b* scales extend from −60 to 60, where a* is negative for green and positive for red, and b* is negative for blue and positive for yellow [38].

### 4.3. Extraction, Purification, and Isolation of Anthocyanins

For HPLC analysis, anthocyanins were extracted from 1 g of finely ground plant material in 1 mL of 1% (*v*/*v*) HCl-methanol for 24 h at 4 °C on a rotating wheel in darkness. Samples were clarified by centrifugation at 13,000× *g* for 15 min at 4 °C, and then 1.5 mL of the supernatant was transferred to autosampler vials. Analysis was conducted on the HPLC and PDA system, which was equipped with a model 1525 binary solvent delivery system (Waters, Milford, CT, USA), an an on-line degasser (Waters, Milford, CT, USA), and a 2707 autosampler (Waters, Milford, CT, USA). The data acquisition and processing were performed by the Breeze software (Waters, Milford, CT, USA). For all of the samples, anthocyanin separations were carried out on a C18 Diamonsil column (250 mm × 4.6 mm, i.d. 5 μm,) (Dikma, Beijing, China). Solvent A was 10% (*v*/*v*) formic acid and solvent B was methanol. The gradient of solvent B was as follows: 0 min, 17%; 1 min, 17%; 9 min, 35%; 20 min, 37%; and 25 min, 100%. The gradient was run at a flow rate of 1 mL/min and a column temperature of 40 °C, with a 5 μL aliquot of sample being injected. Absorbance was measured at 520 nm. Standards were cy3-gal, cy3-glu (Sigma Chemica, St. Louis, MO, USA), cyanidin-3-arabinoside chloride (cy-3-ara) and cyanidin-3-rutinoside chloride (cy-3-rut) (Polyphenols Laboratories, AS, Hanaveien, Bergen, Norway).

### 4.4. Extraction, Purification, and Isolation of the Sugars and Organic Acids

The apple flesh (5.00 g) was used in the extraction. The apples were pooled, and ground to a fine homogenate with a mortar and pestle. The homogenate was diluted to 25 mL with redistilled water, ultrasonically extracted at 90 W for 30 min, cooled to room temperature, and centrifuged at 10,000× g for 10 min. Thereafter, the supernatant was transferred to a 50 mL volumetric flask, the remains were diluted to 15 mL with redistilled water, and ultrasonic extraction was carried out at 90 W for 20 min. Then, the sample was centrifuged at 10,000× g for 10 min and cooled to room temperature. When combined with the supernatant of ultrasonic extraction, the volume was determined to be 50 mL after passing through a 0.45 μm Millipore filter (Millipore Corporation, Bedford, OH, USA). An aliquot (10 mL) of the resultant supernatant was used for HPLC analysis. The sugar (fructose, glucose, sucrose, UDP-galactose, and sorbitol) and organic acid (malic, citric, succinic, oxalic, and tartaric acid) contents were analyzed by HPLC (Waters, Milford, CT, USA). The separation of soluble sugars was carried out using a Sugar Pak TM I column from Waters (300 mm × 6.5 mm) operated at 80 °C. The mobile phase was bi-distilled water, and the flow rate was 0.6 mL/min. The total run time was 25 min, and an RI detector was used for monitoring the soluble sugars, as described by Liu *et al*. [12] with minor changes. Organic acids were analyzed with HPLC using an IC PAK TM ION exclusion column (300 mm × 7.8 mm) (Waters, Milford, CT, USA) associated with a PDA detector set at 210 nm, as described by Liu et al. [12]. The column temperature was set at 50 °C. The elution solvent was 0.01 mM sulfuric acid in bi-distilled water at a flow rate of 0.5 mL/min. The duration of the analysis was 30 min. Sugars and organic acids were expressed as mg/g FW.

### 4.5. Extraction, Purification, and Isolation of Aroma Components

Twenty apples were used in the extraction. The apples were pooled in a vacuum dryer (6 L) and then sealed at 20–22 °C (2 min isothermal). The gases were extracted for 25 min by a polydimethylsiloxane fiber (Supelco, Bellefonte, USA) and then the fibers were desorbed in a gas chromatography (GC) injection liner (SGE, Ringwood, Australia) at 220 °C for 5 min.

The analysis was carried out with an Agilent 6890N/5973N GC-MSD (Santa Clara, CA, USA) and an OV-1701 capillary column (60,000 mm × 250 μm × 0.25 μm). The GC oven temperature was programmed from 35 °C (3 min isothermal) to 60 °C at 10 °C /min (5 min isothermal), from 60–140 °C at 4 °C/min (10 min isothermal), and finally from 140–220 °C at 10 °C/min (10 min isothermal). Helium was the carrier gas at 0.8 mL/min in the constant flow mode. The injector temperature was set to 250 °C, and the split ratio was 1:12. The mass selective detector was equipped with a quadrupole mass analyzer and was operated in the electron ionization mode at 70 eV. The data were evaluated by Mass Selective Detector (MSD) ChemStation D.02.00.275 software (Agilent). The identification of the compounds was carried out by comparing retention times, retention indices and recorded spectra with data known from the literature. The National Institute of Standards and Technology (NIST) library and Wiley library were also consulted [39,40,41]. The percentage data of the total ion current chromatograms were calculated by the area normalization method without applying response factor correction.

### 4.6. Statistical Analysis

Statistical analysis was conducted with Statistical Product and Service Solutions (SPSS) 17.0 (SPSS, Chicago, IL, USA). One-way ANOVA was used for analysis of the anthocyanin, sugar, organic acid, and aroma component concentrations as well as for the color parameters, and differences between the sampling dates were estimated with the Duncan test (*p* ≤ 0.05).

## 5. Conclusions

In this study, developmental changes in the CIE parameters, anthocyanins, soluble sugars, organic acids and aroma components in the flesh fruit of “Starkrimson” were determined during ripening. The results indicated that the changes in anthocyanin contents took place later than the changes in the CIE parameters. Meanwhile, cy3-gal, fructose, sucrose, glucose and malic acid were the main organic compounds, and 1-butanol-2-methyl-acetate, 2-hexenal and 1-hexanol were the most abundant aroma components in the apple skin. Furthermore, rapidly changing soluble sugars and organic acid synchronization took place in the early period, while the changes in the aroma components occurred later, on the basis of fresh weight. This suggested that the production of aroma components might be useful as an index of apple maturity.

## Figures and Tables

**Table 1:** Correlation coefficients between cy3-gal with soluble sugars and organic acids during apple ripening.

Sugars						Organic Acids				
Cy3-gal	fructose	glucose	sucrose	sorbitol	UDP-galactose	oxalic acid	malic acid	citric acid	succinic acid	acetic acid
	0.904 **	0.901 **	0.974 **	-8.75 **	−0.986 **	−0.882 **	−0.777 **	−0.775 **	-0.952 **	−0.952 **

Note: ** indicates *p* < 0.01.

**Table 2:** Main aroma components of “Starkrimson” apple during fruit maturation.

Components Name	Relative Content %									
	95d	100d	105d	110d	115d	120d	125d	130d	135d	140d
Esters										
Formic acid, pentyl ester	–	–	–	–	–	–	–	0.09	–	–
*n*-Propyl acetate	–	–	–	–	–	–	–	–	0.42	0.59
Acetic acid, hexyl ester	1.74	3.51	4.26	5.30	5.71	5,55	5.44	5.36	5.04	4.86
Propanoic acid, butyl ester	–	–	–	–	–	–	–	–	0.41	0.53
Butanoic acid, butyl ester	–	–	–	–	0.05	0.06	0.17	0.34	0.45	0.58
Hexanoic acid, ethyl ester	–	–	–	–	1.13	1.08	1.04	1.54	0.91	0.80
Hexanoic acid, propyl ester	–	–	0.20	0.31	0.32	0.43	0.57	0.65	0.68	0.56
Hexanoic acid, butyl ester	–	–	–	–	0.15	0.54	0.84	0.91	1.29	2.66
Isopentyl hexanoate	–	–	–	–	0.05	0.14	0.27	0.34	0.51	0.72
Hexanoic acid, hexyl ester	–	–	–	–	0.06	0.24	0.43	1.04	1.45	1.74
1-Butanol, 2-methyl-, acetate	*–*	0.10	0.14	0.15	0.18	0.28	0.44	2.02	7.45	10.11
2-Hexen-1-ol, acetate	5.57	8.67	9.19	9.65	9.85	10.48	9.39	8.14	5.67	4.35
Butyl 2-methylbutanoate	–	–	–	–	–	–	0.41	0.54	0.67	1.56
Butanoic acid, 2-methyl-, hexyl ester	–	–	–	–	0.11	0.49	0.84	1.02	2.16	3.01
Butanoic acid, 2-methyl-, 2-methylbutyl ester	–	–	–	–	–	–	0.42	0.51	0.62	0.67
Aldehydes										
Hexanal	5.58	6. 73	6.84	7.76	7.82	8.02	9.18	11.05	12.22	13.28
2-Hexenal, (*E*)-	0.81	0.42	0.36	0.28	0.32	0.42	0.76	1.27	1.24	1.21
2-Hexenal,(*Z*)-	69.61	53.34	46.11	39.75	35.06	33.06	32.64	32.04	26.53	23.24
3-Hexenal, (*Z*)-	1.85	1.32	1.17	0.94	0.74	0.63	0.65	0.61	0.64	0.68
2,4-Hexadienal, (*E*,*E*)-	0.66	1.09	1.30	1.54	1.17	0.88	0.81	0.68	0.60	0.53
Benzeneacetaldehyde	1.07	0.75	0.63	0.52	0.28	0.15	0.08	0.04	–	–
Alcohols										
Ethanol	0.06	0.23	0.27	0.32	0.31	0.30	0.29	0.29	0.25	0.21
1-Propanol	–	–	–	–	–	–	0.17	0.26	0.32	0.40
1-Butanol	–	0.07	0.25	1.04	1.07	1.08	1.12	1.25	1.81	2.30
1-Hexanol	–	0.47	0.86	1.22	1.38	3.22	4.07	5.14	7.15	8.11
1-Penten-3-ol	0.38	0.75	0.86	1.03	1.09	1.01	0.83	0.48	0.48	0.47
2-Hexen-1-ol, (*E*)-	11.77	21.57	26.85	29.15	30.72	29.23	24.68	19.14	14.96	11.62
2-Penten-1-ol, (*Z*)-	–	0.31	0.70	1.12	1.08	0.81	0.87	0.96	0.64	0.33
3-Hexen-1-ol, (*Z*)-	0.14	0.62	0.92	1.33	1.08	0.92	0.86	0.31	0.15	–
1-Butanol, 2-methyl-	–	0.06	0.06	0.07	0.09	0.12	0.73	0.89	1.14	2.09
Others										
α-Farnesene	–	–	–	–	–	0.19	0.42	0.65	0.58	0.34
Butanoic acid, 2-methyl-	–	–	–	–	–	–	2.14	2.42	2.49	2.21
n-Hexadecanoic acid	–	–	–	–	0.17	0.61	0.46	–	–	–

volatiles only listed relative content more than 0.5%.–: Not found or does not exist.
